# Modularity detection in protein-protein interaction networks

**DOI:** 10.1186/1756-0500-4-569

**Published:** 2011-12-29

**Authors:** Tejaswini Narayanan, Merril Gersten, Shankar Subramaniam, Ananth Grama

**Affiliations:** 1 Department of Electrical and Computer Engineering, University of California, San Diego, USA; 2 Graduate Program in Bioinformatics and Systems Biology, University of California, San Diego, USA; 3 Department of Bioengineering, University of California, San Diego, USA; 4 Department of Computer Science, Purdue University, West Lafayette, IN, USA

## Abstract

**Background:**

Many recent studies have investigated modularity in biological networks, and its role in functional and structural characterization of constituent biomolecules. A technique that has shown considerable promise in the domain of modularity detection is the Newman and Girvan (NG) algorithm, which relies on the number of shortest-paths across pairs of vertices in the network traversing a given edge, referred to as the *betweenness *of that edge. The edge with the highest betweenness is iteratively eliminated from the network, with the betweenness of the remaining edges recalculated in every iteration. This generates a complete dendrogram, from which modules are extracted by applying a quality metric called *modularity *denoted by *Q*. This exhaustive computation can be prohibitively expensive for large networks such as Protein-Protein Interaction Networks. In this paper, we present a novel optimization to the modularity detection algorithm, in terms of an efficient termination criterion based on a *target edge betweenness *value, using which the process of iterative edge removal may be terminated.

**Results:**

We validate the robustness of our approach by applying our algorithm on real-world protein-protein interaction networks of *Yeast, C.Elegans *and *Drosophila*, and demonstrate that our algorithm consistently has significant computational gains in terms of reduced runtime, when compared to the NG algorithm. Furthermore, our algorithm produces modules comparable to those from the NG algorithm, qualitatively and quantitatively. We illustrate this using comparison metrics such as module distribution, module membership cardinality, modularity *Q*, and Jaccard Similarity Coefficient.

**Conclusions:**

We have presented an optimized approach for efficient modularity detection in networks. The intuition driving our approach is the extraction of holistic measures of centrality from graphs, which are representative of inherent modular structure of the underlying network, and the application of those measures to efficiently guide the modularity detection process. We have empirically evaluated our approach in the specific context of real-world large scale biological networks, and have demonstrated significant savings in computational time while maintaining comparable quality of detected modules.

## Background

The problem of modularity detection in networks has received considerable attention in recent literature [1-5]. Specifically, in the context of biological networks, identification of modules enables functional annotation of constituent biomolecules, discovery of targets for therapeutic intervention and screening etc. More generally, modular decomposition provides us with a higher-level understanding of the organization of networks and also serves as the basis for other network analysis tasks, such as hierarchical alignment, modular evolution, and orthology.

There are three primary approaches to modularity detection: (i) top down (or divisive) techniques, in which a series of network partitions hierarchically decompose a network into modules, (ii) bottom up (or agglomerative) techniques, in which modules are constructed by adding elements to an initial seed, and (iii) force directed methods, in which suitably designed parameters drive nodes belonging to the same module to spatially proximate regions of space. There have also been investigations focused on relating various classes of methods [6].

### Newman and Girvan algorithm

One such divisive technique of interest is the Newman and Girvan (NG) algorithm [1], which uses the notion of *edge-betweenness*, a metric that has received considerable recent research interest in the domain of modularity detection. Edge-betweenness is typically computed as the number of (pair-wise) shortest paths that traverse an edge in a network. This notion, which was first introduced by Anthonisse [7], can be used to compute modules by repeatedly identifying and eliminating the edge with highest betweenness. Note that since the elimination of a single edge (especially one with high betweenness) may cause significant perturbations to the shortest paths, the edge-betweenness of the remaining edges must be recomputed after each edge-elimination.

The output from the NG algorithm is a complete dendrogram, which decomposes a given graph down to individual nodes. Modules are extracted from this dendrogram by applying a quality metric called *modularity *(*Q*), which is defined as follows:

$$Q\;=\;\underset{i}{\sum }\left(e_{ii}-a_{i}^{2}\right)\;=\;Tr\left(e\right)\;-∥e^{2}∥$$

where, *e *is a *k *× *k *symmetric matrix whose element *e_ij _*is the fraction of all edges in the network that link vertices in module *i *to vertices in module *j*;*k *is the number of modules in the network;

*Tr*(*e*) = ∑*_i _*e*_ii_*, is the trace of *e*, which represents the fraction of edges in the network that connect vertices in the same module;

*a_i _*= ∑*_j _*e*_ij_*, are the row (or column) sums, which represent the fraction of edges that connect to vertices in module *i*;

||*E*|| denotes the sum of the elements of matrix *E*.

We observe that, in a network in which edges fall between vertices without regard for the modules they belong to, *e_ij _*= *a_i_a_j_*.

The *Q *value measures the fraction of the edges that connect vertices within the same module minus the expected value of the same quantity in the network. If the number of intra-modular edges is no better than random, we get *Q *= 0. Values approaching *Q *= 1, which is the maximum, indicate strong modular structure [1]. In practice, *Q *values for such networks with strong modular structure typically fall in the range from about 0.3 to 0.7. The modular decomposition of the network (from the dendrogram) with maximum *Q *value is considered to be the best split by the NG algorithm.

While the computation of modules using the NG algorithm has been shown to perform well in terms of quality of modules, its computational cost can be significant (particularly for large networks such as biological networks). This cost, in part, stems from repeated edge betweenness computations. Furthermore, a level of refinement in the output dendrogram to the individual nodes, is typically unnecessary from an application standpoint, often un-informative, and computationally expensive. Finally, the dendrogram requires additional post-processing to identify suitable modules based on quality measures associated with the modules. Computing the quality of each module corresponding to every node in the dendrogram is itself expensive. A stopping criterion that identifies a near-optimal point at which the process of iterative edge-removal may be terminated would significantly reduce the time and space complexity of the NG algorithm.

The problem of terminating divisive clustering is an important one, especially when the clustering method is itself expensive. A number of other approaches have been proposed--including use of *p *values of clusters as termination criteria [8]. However, each of these methods assumes models for underlying data, or specific properties for quality measures applied to modules. For example, the divisive partitioning technique of Koyuturk et al. [8] stops the partitioning process when the *p *value of a module is lower than a user-specified threshold. This does not guarantee that the optimal *p *value modules are found. Similarly, for data-sets for which precise models are not available, estimation of number of clusters is difficult. Neither class of techniques is directly applicable for divisive partitioning based on the NG algorithm.

In this paper, we experimentally derive an optimized termination criterion for the NG algorithm (which we call the *target edge-betweenness*), based on initial values of edge-betweenness computed over the input network. In particular, we define the *target edge-betweenness *to be the *geometric mean *of edge-betweenness values of all edges in the input network (and hence refer to our algorithm as the *Gmean algorithm *in the discussion below). A detailed description of our algorithm is included in the Methods section.

## Results and discussion

There are two computational problems with the NG algorithm:

1. The iterative removal of edges (preceded by recalculation of edge betweenness in every iteration) is performed until all the edges are removed, leading to a time complexity of *O *(*ne^2^*) for a network of *n *vertices and *e *edges (using Brandes' algorithm, assuming connected networks as inputs). This computation becomes prohibitively expensive in the context of large biological networks.

2. The modularity *Q *is calculated for every partition of a network in the dendrogram. This is necessary for determining optimal splits.

The Gmean algorithm directly addresses these overheads in two fundamental ways: it terminates the process before all edges are removed, thus significantly reducing the first overhead. Since the termination criterion is computed just once (at the start of the algorithm), and does not rely on repeated *Q *value computations, we eliminate the second overhead altogether.

Furthermore, we demonstrate that our algorithm results in modules with *Q *values comparable to the maximum *Q *value from the NG algorithm--thus maintaining the quality of the identified modules, while significantly reducing runtime. We also use the *Jaccard Similarity Coefficient *(a measure of *similarity *between two sample sets) to show that the resulting modules from both the approaches are similar.

We validate our approach on the networks summarized in Table 1. For each of the networks, we eliminate multiple edges between pairs of nodes, self-loops, and mirrored edges. Thus, the final number of edges/interactions considered is shown in #Edges (Network considered).

**Table 1:** Summary of Networks that were used to validate our approach

Network	Source	#Vertices [Original Network]	#Edges	
			
			Original Network	Network Considered
C.Elegans	[9]	453	4596	2025

Yeast*	[10]	3654	15316	9946

Drosophila	[10]	7666	25649	25433

* The entire Yeast network contains 160,566 interactions. We restrict the dataset to interactions determined by Co-purification or Yeast Two-hybrid experiments. This yields a network of 15,316 interactions

**Figure 1:**
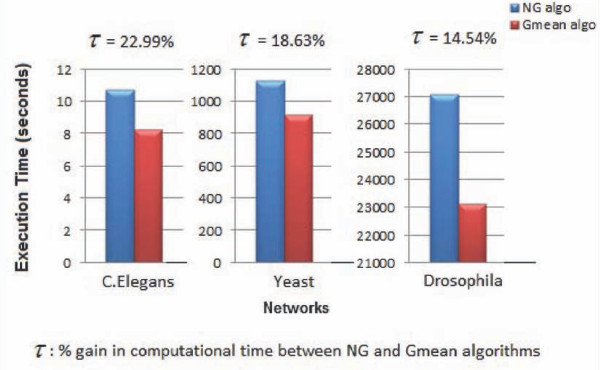
**Comparison of Runtimes for NG and Gmean algorithms for C.Elegans, Yeast and Drosophila networks**.

We perform our experimental evaluation using a parallelized approach [11] to implement the NG and Gmean algorithms. Our results (as shown in Figure 1) demonstrate excellent performance in terms of efficiency on moderate machine configurations (tens of processors).

### Comparison of computational efficiency

For a specific network under consideration, let *RT_NG _*and *RT_Gmean _*denote the execution times for the NG and Gmean algorithms respectively. We define the percentage gain in computational time (τ) between the NG and Gmean algorithms, as follows:

$$\tau \text{ = }\;\frac{\text{R}{\text{T}}_{\text{NG}}-\text{R}{\text{T}}_{\text{Gmean}}}{\text{R}{\text{T}}_{\text{NG}}}\;\times \;100$$

We observe significant and consistent savings in computational cost with our proposed optimization (for the networks in our biological test bed under consideration). Figure 1 presents a comparison of the execution times for the NG and Gmean algorithms.

### Comparison of module size and distribution

**Figure 2:**
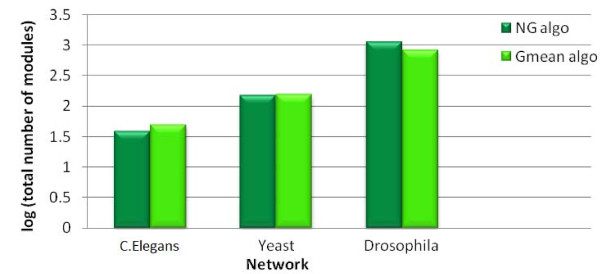
**Log scale comparison of total number of modules identified by NG and Gmean algorithms for C.Elegans, Yeast and Drosophila networks**.

**Figure 3:**
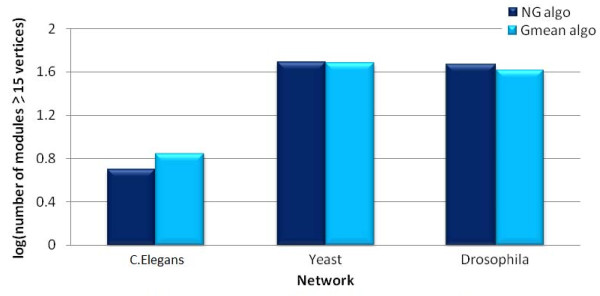
**Log scale comparison of number of modules with at least 15 vertices identified by NG and Gmean algorithms for C.Elegans, Yeast and Drosophila networks**.

In Figures 2 and 3, we present a broad quantitative comparison of the size and distribution of modules produced using the Gmean and NG algorithms. In particular, we observe that, for all the three networks under consideration, the total number of modules produced by the two algorithms is comparable.

### Comparison of modularity

**Figure 4:**
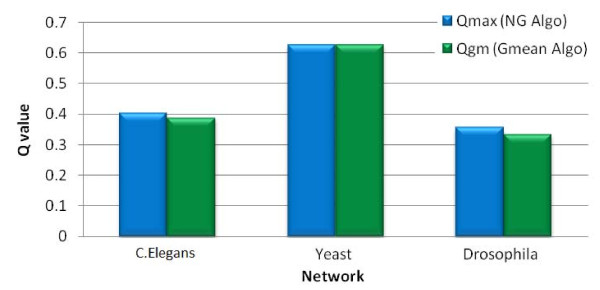
**Comparison of Modularity (Q) values from NG and Gmean algorithms for C.Elegans, Yeast and Drosophila networks**.

In addition to quantitatively comparing and demonstrating that the modules resulting from our algorithm are comparable in number and distribution to the modules resulting from the NG algorithm, we also present a qualitative validation that the results are indeed statistically similar in terms of *quality *of the modules produced using the modularity value *Q*. Figure 4 shows the modularity value comparison for the set of modules produced by both the algorithms, for the different networks considered in this paper. We note that for all networks under consideration, our algorithm identifies modules with very similar modularity values as the NG algorithm.

### Comparison of Jaccard similarity coefficient

*Jaccard Similarity Coefficient *or the *Jaccard Index *is a statistic used for comparing the similarity and diversity of sample sets. The Jaccard Index measures similarity between two sample sets (say *A *and *B*), and is defined as the size of the intersection divided by the size of the union of the sample sets:

$$J\left(A,B\right)\;=\;\frac{|A\cap B|}{|A\cup B|}$$

The Jaccard Index is 1 if the two sample sets are exactly identical, and is equal to 0, if they have no overlap at all.

We use this metric to show the similarity of the modules produced as the output by the NG and the Gmean algorithms. Specifically, we consider the modules produced by the algorithms as sample sets constituted by vertices and calculate the Jaccard Indices *J *(*A,B*) for all pairs of modules *A *and *B *(one from the output of each algorithm).

We define the percentage similarity score (λ) as the following:

$$\lambda \;=\;\frac{\sum J\left(A,B\right)}{\sum J\left(A,B\right)*}\;\times \;100$$

where *J *(*A,B*) is the Jaccard Index for the modules *A *and *B*, one from the output of each algorithm;

*J *(*A,B*)* is the *ideal *Jaccard Index for the modules *A *and *B*, one from the output of each algorithm (note that *J *(*A,B*)* = 1, corresponding to perfect match, when the two modules *A *and *B *are exactly identical);

Σ is the summation over all pairs of modules, one from the output of each algorithm.

Table 2 shows the percentage similarity values for the modules produced by the two algorithms for all the networks considered. We observe that the modules produced by the two algorithms demonstrate a high degree of similarity.

**Table 2:** Summary of % similarity for biological networks considered

	C.Elegans	Yeast	Drosophila
**Σ ***J(A,B)*	4.5472	47.973	40.5089

**Σ ***J(A,B)*	5	48	46

λ	90.94%	99.94%	88.06%

## Conclusions

In this paper, we have proposed a novel termination criterion for efficient modularity detection in networks. The intuition driving our approach is the extraction of holistic measures of centrality from graphs, which are representative of inherent modular structure, and the application of those measures to efficiently guide the modularity detection process. We have empirically evaluated our approach against existing techniques for modularity detection in the context of biological networks, and have demonstrated significant savings in computational time while maintaining comparable quality of detected modules.

## Methods

### Existing NG method

In the NG algorithm, the edge-betweenness is computed for each edge in the network under consideration. The edge with the maximum edge-betweenness is identified and eliminated, followed by a recalculation of the edge-betweenness values of all the remaining edges in the resultant network. This process is iteratively repeated till no edges are remaining, thus generating a complete dendrogram which is then traversed to identify the partition with best modularity value *Q*.

### Proposed Gmean method

**Figure 5:**
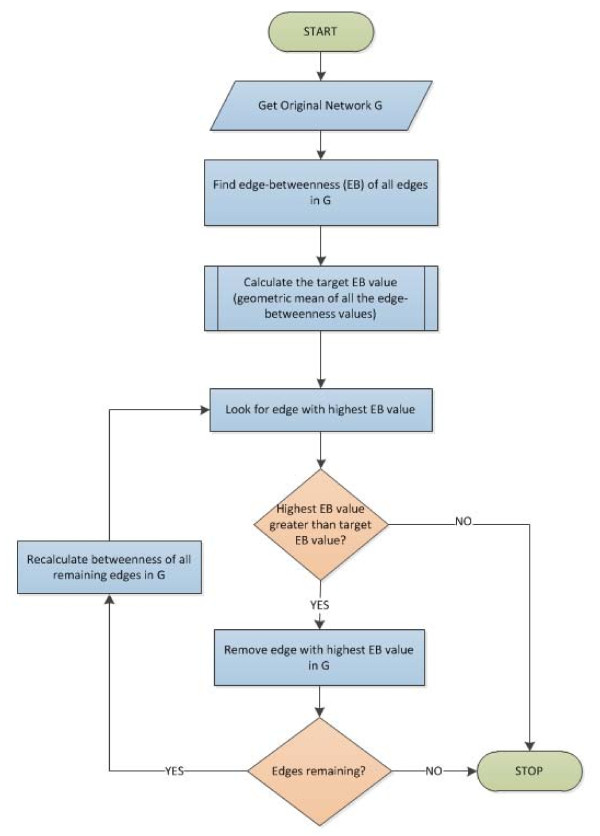
**Flow diagram illustrating the general framework of the proposed Gmean algorithm**.

Figure 5 presents a flow diagram that illustrates the general framework of the proposed Gmean algorithm. Our motivation is to compute a *target edge betweenness T *that is used to determine termination of the algorithm. In particular, we propose that the recalculation of edge-betweenness and removal of the edges be stopped when the edge to be removed has a betweenness value less than *T*. More intuitively, we propose that for an edge to be considered to be an inter-modular edge, it must have betweenness value of at least *T*.

Based on extensive experimentation, we propose the following definition of *T*:

T=G(e)

where *G *(*e*) is the geometric mean (*gmean*) of edge-betweenness values of all edges in the input network. Validation on real networks shows that this choice serves as a robust and high-quality termination criterion. Specifically, as stated in the results section, this choice produces a set of modules comparable in quality and quantity to those produced by the NG algorithm. We show this for a number of biological networks of interest. All biological network data used for the experimental study are from publicly available data sources [9,10].

## List of abbreviations

C.Elegans: Caenorhabditis elegans; gmean: Geometric Mean.

## Competing interests

The authors declare that they have no competing interests.

## Authors' contributions

TN investigated the problem of modularity detection and associated literature, proposed the optimization to the existing Newman and Girvan algorithm, and empirically evaluated the approach. MG helped with refining the proposed optimization and perform quantitative comparison. SS and AG provided guidance relative to the theoretical and practical aspects of designing/evaluating the algorithm. All authors read and approved the final manuscript.
